# Evaluation of water quality based on a machine learning algorithm and water quality index for the Ebinur Lake Watershed, China

**DOI:** 10.1038/s41598-017-12853-y

**Published:** 2017-10-09

**Authors:** Xiaoping Wang, Fei Zhang, Jianli Ding

**Affiliations:** 1 College of Resources and Environment Science, Xinjiang University, Urumqi, 830046 Xinjiang China; 2 Key Laboratory of Oasis Ecology, Xinjiang University, Urumqi, 830046 Xinjiang China; 3 Key Laboratory of Xinjiang wisdom city and environment modeling Urumqi, Urumqi, 830046 Xinjiang China

## Abstract

The water quality index (WQI) has been used to identify threats to water quality and to support better water resource management. This study combines a machine learning algorithm, WQI, and remote sensing spectral indices (difference index, DI; ratio index, RI; and normalized difference index, NDI) through fractional derivatives methods and in turn establishes a model for estimating and assessing the WQI. The results show that the calculated WQI values range between 56.61 and 2,886.51. We also explore the relationship between reflectance data and the WQI. The number of bands with correlation coefficients passing a significance test at 0.01 first increases and then decreases with a peak appearing after 1.6 orders. WQI and DI as well as RI and NDI correlation coefficients between optimal band combinations of the peak also appear after 1.6 orders with R^2^ values of 0.92, 0.58 and 0.92. Finally, 22 WQI estimation models were established by POS-SVR to compare the predictive effects of these models. The models based on a spectral index of 1.6 were found to perform much better than the others, with an R^2^ of 0.92, an RMSE of 58.4, and an RPD of 2.81 and a slope of curve fitting of 0.97.

## Introduction

Water shortage problems in semi-arid areas have become more and more serious in recent years^1–4^. Recent studies show that a lack of water resources could affect nearly 5.5 billion people in 10 years^5^. Severe water shortages and large volumes of sewage render river and lake water pollution issues serious in arid areas^6,7^. The water quality of rivers and lakes is becoming central to human and economic development. Therefore, the evaluation and estimation of water quality levels is essential for societal and economic development^8^.

With advances in space information science and with an increasing use of computer applications in recent years, remote sensing has become a useful tool of surface parameter monitoring^9,10^. It allows one to monitor large scale water bodies that suffer from qualitative problems more effectively. Via remote sense, an optical reflectance sensor was used in this study. Optical sensor systems use sunlight as a source of light and are equipped with light-emitting components that provide radiation in specific band regions^11,12^. The optical sensors generate hyperspectral information on water quality levels in the visible and near-infrared ranges. Some studies have evaluated relationships between hyperspectral reflectance wavebands and water quality parameters.

Studies on surface water spectral features and modified model methods have shown that it is possible to perform water quality parameter monitoring by applying remote sensing technologies to more water quality variables with higher precision. Single water quality parameters such as chlorophyll-a, total suspended solids, turbidity levels, transparency levels, levels of dissolved organic matter, chemical oxygen demand, biological oxygen demand, etc., have been widely estimated through remote sensing technology monitoring^10,13–19^. Although estimated models of water quality parameters are relatively accurate, they generate uncertain results because water environments are complex and changeable. Therefore, a water body spectrum is shown for the entire water environment and is not a single water quality parameter. Many scholars have developed estimation models of a single water quality parameter based on water body spectrum data^10,17,18^. Thus, estimation models of a single water parameter introduce a certain level of uncertainty. From such analyses, a water quality index that reflects the entire water environment should be developed to evaluate the entire water environment.

Several methods for evaluating the water quality levels of rivers and lakes have been introduced^20,21^. Therefore, a good water quality assessment method should not only accurately reflect spatial variations in water quality, but should also conveniently to quickly monitor water quality levels. The water quality index (WQI)^22–26^ is used for the water quality assessment of drinking water source by the Ministry of Water Resources, Monitoring and Evaluation Center of Water Environment. The WQI was initially proposed by Horton^27^ and Brown *et al*.^28^. Since then, various methods for the calculation of the water quality index (WQI) have been designed by several authors^29–31^. WQI is a mathematical instrument used to transform large quantities of water characterization data into a single value that represents the water quality level and that reflects overall water quality levels^32^. However, while WQI methods can assess the water quality of a single sample, they are not easily able to identify spatial or temporal variations in water quality, which are vital to the comprehensive assessment and management of surface water quality. These difficulties associated with successive and integrated sampling have become a significant obstacle to the monitoring and management of water quality, and remote sensing technologies make up for shortcomings of spatial and temporal variations. The establishment of a water quality index that can be widely used for environmental management and that is easy to calculate, to master and to use to meet remote sensing monitoring requirements is explored in this study.

The main objectives of this study are (i) to create a water quality index (WQI) for surface water quality evaluation and classification in arid areas and to create a WQI map via GIS (ii) to extract sensitive wave bands and build a spectral index (RI, DI, NDI) that is significantly related to the water quality index, (iii) to establish an estimation model of the water quality index (WQI) based on the spectral index (RI, DI, NDI), to develop sensitive wave bands and a Support Vector Regression Model (SVR) for dry areas, and (iv) to estimate the accuracy of the model relative to WQI values. We not only assess water quality levels using the WQI for a semi-arid area, but we also develop a new algorithm that can estimate the WQI via remote sensing techniques.

## Results and Analysis

### Statistical analysis of the water quality index

A summary of water quality observations for Ebinur Lake Watershed surface water of the Boertala River, the Jing River, the Akeqisu-Kuitun River (A-KR) and artificial reservoirs (RES) for October of 2016 is presented in Table 1. At different water quality levels, (pH) levels varied considerably from 7.62–8.46 spanning one order of magnitude with a mean value of 7.97 and Coefficient of Variation of 12.29%. Concentrations of TDS also experienced varied considerably from 81.4 mg/L–9470 mg/L with a mean value of 728.88 mg/L and with a Coefficient of Variation of 19.2%. TDS values of the Ebinur Lake Watershed were found to be lower and strongly variable and most likely because upstream reservoirs of the Bolatala and Jing Rivers serve as a settling watershed. Ca levels of the four rivers were found to be similar and to range from low to moderate (42.8 mg/L–1082.16 mg/L) with an average value of 161.1 mg/L and a Coefficient of Variation of 144.02% and characterized by strong variations in the Ebinur Lake Watershed. (TN), (BOD_5_) and DO values were found to be similar in the Ebinur Lake Watershed with average values of 1.54 mg/L, 2.26 mg/L, and 29.12 mg/L respectively with a low Coefficient of Variation of (<100%) and less variation. Concentrations of NH_3_^+^-N were also highly variable at 0.01 mg/L–9.21 mg/ with a mean value of 0.62 mg/L and a Coefficient of Variation of 316.79%. (COD) and TP values exhibit similar trends with Coefficient of Variation values of between 100% and 200%. For metal indicators, concentrations of (Iron), (Mg), (Na), (Copper), (Zinc) and (Volatile phenol) are similar with Coefficients of Variation varying considerably between 100% and 200%. In addition, the Coefficient of Variation for Mg was measured at 318.56. (HCO_3_^−^) and varies considerably from 89.94–24324.13 spanning one order of magnitude with a mean value of 1,419.31 mg/L and with a Coefficient of Variation of 358.74% that is highly variable. Concentrations of SO_4_^2−^ also varied considerably from 4.8 mg/L–8424 mg/L with a mean value of 961.88 mg/L and a Coefficient of Variation of 172.28%. SO_4_^2−^ levels in the Ebinur Lake Watershed were found to be lower and strongly variable and most likely due to the presence of the Boertala and Jing River reservoirs upstream, which serve as a settling watershed. (PO_4_^3−)^ was found to vary considerably from 0–1.7, spanning one order of magnitude with a mean value of 0.2237 mg/L and highly variable Coefficient of Variation of 153.57%. (Cr) was found to vary considerably from 0.01–0.16, thus spanning one order of magnitude with a mean value of 0.0283 mg/L and a highly variable Coefficient of Variation of 102.47%. In short, the water quality index changes considerably in this watershed while pH, DO and TDS values change less. Water quality levels thus vary considerably in the watershed.

**Table 1:** Summary of water quality observations of the Ebinur Lake Watershed for October of 2016.

Water quality index	Data set	Min value	Max value	Mean value	Standard deviation value	Coefficient of Variation/%
pH	48	7.62	8.46	7.97	0.98	12.29
TN	48	0.24 mg/L	7.06 mg/L	1.54 mg/L	1.28 mg/L	82.84
BOD5	48	0.80 mg/L	7.80 mg/L	2.64 mg/L	1.39 mg/L	52.66
TP	48	0.01 mg/L	0.99 mg/L	0.22 mg/L	0.25 mg/L	116.15
NH_3_^+^-N	48	0.01 mg/L	9.21 mg/L	0.62 mg/L	1.95 mg/L	316.76
COD	48	0.70 mg/L	174 mg/L	136.70 mg/L	347.57 mg/L	254.25
Iron	48	0.01 mg/L	1.65 mg/L	0.15 mg/L	0.27 mg/L	179.85
Copper	48	0.01 mg/L	1.98 mg/L	0.33 mg/L	0.51 mg/L	157.09
Zinc	48	0.01 mg/L	3.31 mg/L	0.45 mg/L	0.59 mg/L	133.82
DO	48	1.40 mg/L	10.4 mg/L	6.18 mg/L	1.80 mg/L	29.12
Volatile phenol	48	0.01 mg/L	5.43 mg/L	0.65 mg/L	1.28 mg/L	194.71
TDS	48	89.41 mg/L	9470 mg/L	728.89 mg/L	142.35 mg/L	19.52
Ca	48	42.80 mg/L	1082.16 mg/L	161.15 mg/L	232.11 mg/L	144.02
Mg	48	8.50 mg/L	3766.5 mg/L	210.54 mg/L	670.73 mg/L	318.56
Na	48	2.6 mg/L	6750 mg/L	479.74 mg/L	1353.76 mg/L	282.18
Cl^−^	48	17.25 mg/L	8838.57 mg/L	555.17 mg/L	1761.13 mg/L	317.22
HCO_3_^−^	48	89.94 mg/L	24324.13 mg/L	1419.31 mg/L	5091.52 mg/L	358.74
SO_4_^2−^	48	4.803 mg/L	8424 mg/L	961.88 mg/L	1657.12 mg/L	172.28
PO_4_^3−^	48	0 mg/L	1.7 mg/L	0.233 mg/L	0.3589 mg/L	153.57
Cr	48	0.01 mg/L	0.16 mg/L	0.028 mg/L	0.029 mg/L	102.47

### Assessment of water quality based on the WQI

In this study, the quality of the Ebinur Lake Watershed surface water was evaluated. To assess the water quality of the river, the WQI method was used. pH, HCO_3_^−^, TP, TN, BOD, NH_3_^+^-N, Iron, Copper, Zinc, Volatile phenol, DO, TDS, Cl^−^, SO_4_^2−^, Na, Ca, Mg, COD, PO_4_^3−^ and Cr values were taken into account for the calculation of WQI values for each sampling location in the Ebinur Lake Watershed in October of 2016. Analysis results for all 48 sampling points were used for quality evaluations. Furthermore, World Health Organization^33^ limits were used for the calculations. Distribution maps of the water quality parameters (pH, HCO_3_^−^, TP, TN, BOD, NH_3_^+^-N, Iron, Copper, Zinc, Volatile phenol, DO, TDS, Cl^−^, SO_4_^2−^, Na, Ca, Mg, COD, PO_4_^3−^ and Cr) and a WQI map for the river were prepared using Geographic Information System (GIS) techniques and are presented in Fig. 4 and Table 2.

**Table 2:** Assessment of water quality using the WQI.

	Parameters	WHO standards (2008)	Weight (Wi)	Relative weight (Wi)
1	pH	6.80–8.50	4.00	0.072
2	TDS	450.00	1.00	0.018
3	COD	15.00	4.00	0.072
4	BOD5	3.00	5.00	0.091
5	TP	0.10	3.00	0.054
6	TN	0.50	3.00	0.054
7	NH_3_^+^-N	0.50	3.00	0.054
8	V.P.	0.02	4.00	0.072
9	Ca	300.00	2.00	0.036
10	Mg	30.00	2.00	0.036
11	Na	200.00	2.00	0.036
12	Fe	0.30	1.00	0.018
13	Cu	1.00	1.00	0.018
14	Zn	1.00	2.00	0.036
15	HCO_3_^−^	/	3.00	0.054
16	Cl	250.00	3.00	0.054
17	SO_4_^2−^	250.00	4.00	0.072
18	PO_4_^3−^	50.00	5.00	0.091
19	Gr	0.05	1.00	0.018
20	DO	6	1	0.018
			58	1

**Figure 1:**
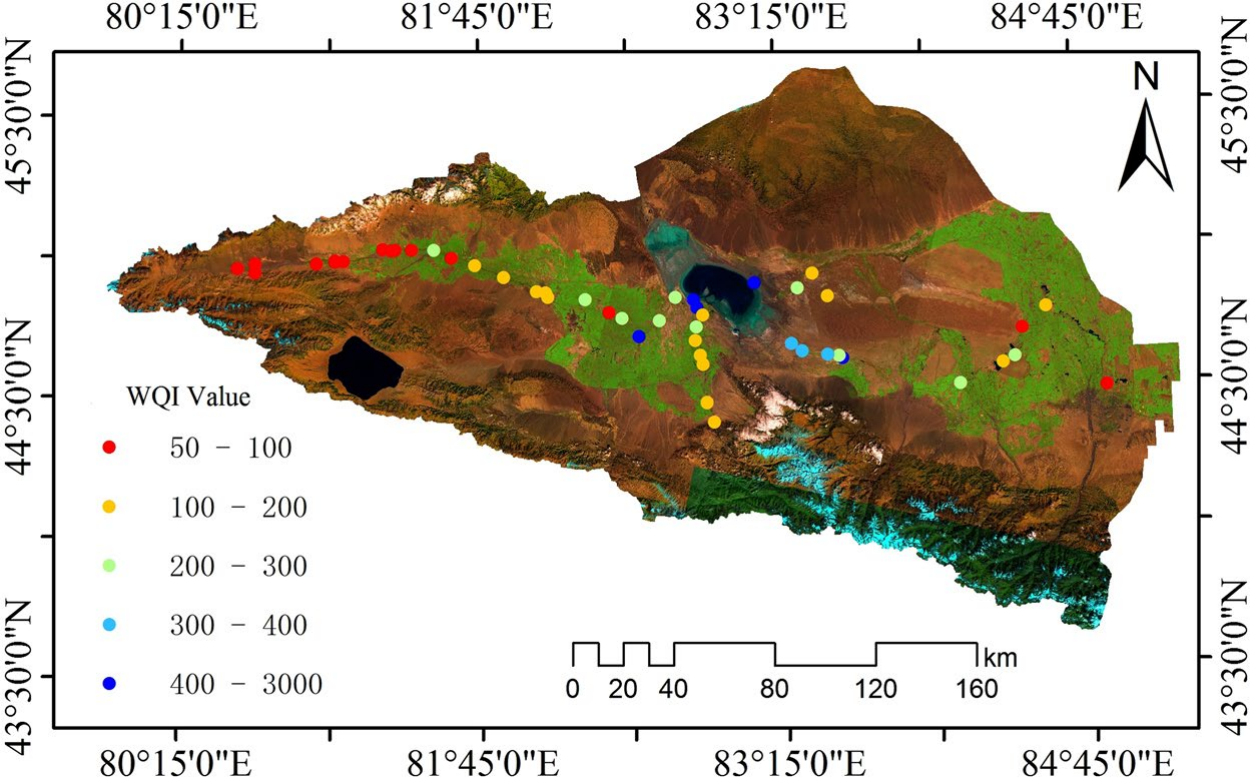
Spatial characteristics of the WQI for the Ebinur Lake Watershed (Map by ArcGIS10.2.2 (http://www.esri.com/software/arcgis)).

Spatially, water quality index (WQI) levels are high for most areas of the Boertala River downstream from Ebinur Lake and (Fig. 1) and occupy the V category. This water is unsuitable for drinking. The highest value of 438 is observed for the Kuitun River. As this water body is located in the town of Tuotuo, the effects of human factors are severe, and water quality levels in this river are poor. Therefore, as water quality levels worsen, WQI levels increase. The best levels of water quality for the Ebinur Lake Watershed are found in the upper reaches of the Bortala River. Its WQI value is less than 100 (I grade water quality) and is suitable for drinking. Poor water quality levels are observed for midstream reaches of Boertala River of Wenquan County where the effects of human factors are severe and where water quality levels have resulted in mutations and in the development of water quality index anomalies. From an ecological perspective, the ecological environment of Ebinur Lake is the worst in the watershed. Rivers originate from mountains surrounding the watershed where the ecological environment is superior to that of Ebinur Lake.

### Hyperspectral characteristics of surface water

**Figure 2:**
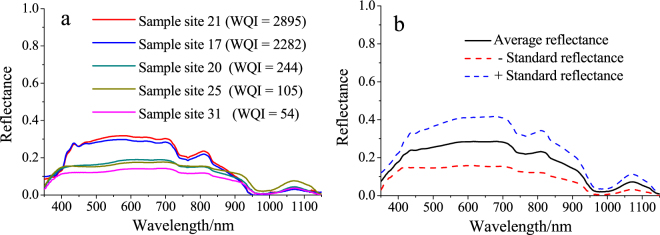
Spectral curves of water in different rivers (Map by Origin 9.1 (http://www.originlab.com/software)).

Figure 2 (a) shows how on the basis of the river areas described above, 48 water samples were classified into 5 categories and spectral plots of each category were averaged as a representative spectral curve of this water quality level (Fig. 2a). Five spectral plots of similar shapes were identified with two pronounced absorption features located at approximately 700 and 950 nm. Of the five categories, sample site 31 exhibited lowest reflectance and a location slightly downstream exhibited the highest reflectance. Sample site 21 presented the highest reflectance value. This sample site is located in the downstream area of the river (into the lake). For each class, an average spectrum was calculated (Fig. 2a), and the plots show reflectance curves of two deep absorption regions at 750 and 980 nm and several weak absorption regions at approximately 452 nm, 703 nm, and 850 nm. It was easy to identify differences in water quality at roughly 700–720 nm and 1,070 nm of the peak. Average and standard values are shown in Fig. 2(b) with no outliers and a normal distribution.

### Correlations between the water quality index and spectra

**Figure 3:**
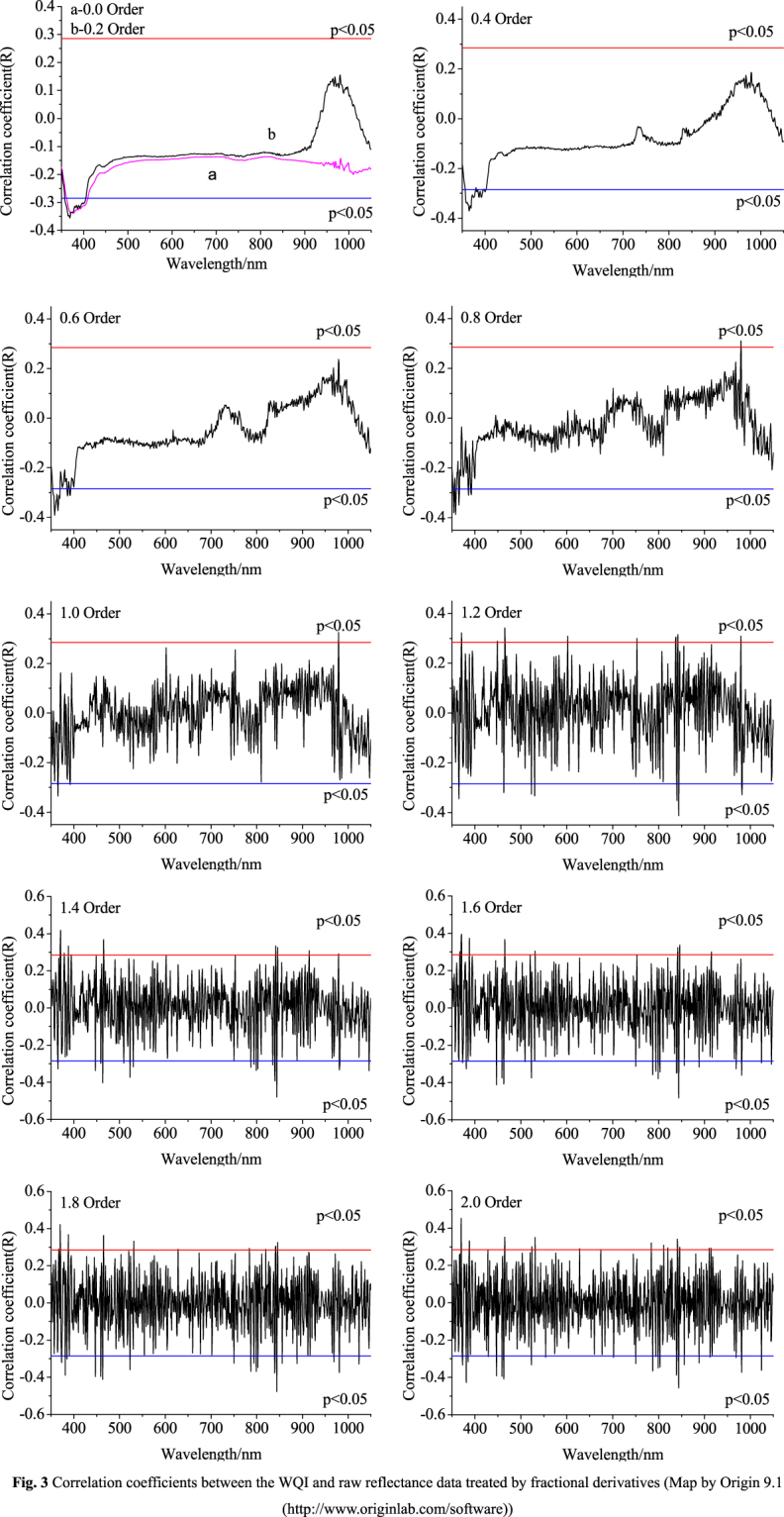
Correlation coefficients between the WQI and raw reflectance data treated by fractional derivatives (Map by Origin 9.1 (http://www.originlab.com/software)).

Sensitive wave band selection is central to constructing a water quality index (WQI) estimation model, and correlation coefficients for the water quality index (WQI) and spectral reflectance (single wave bands) are usually used to identify water quality index bands (sensitive wave bands). All correlation coefficients between the water quality index (WQI) and raw reflectance data treated based on fractional derivatives (0 order, 0.2 order, 0.4 order, 0.6 order, 0.8 order, 1.0 order, 1.2 order, 1.4 order, 1.6 order, 1.8 order, and 2.0 order) were tested with a significance level of 0.01 (|r| = 0.24 or above). Spectral curves of correlation coefficients of the raw reflectance and of raw reflectance data treated by fractional derivatives (0 order, 0.2 order, 0.4 order, 0.6 order, 0.8 order, 1.0 order, 1.2 order, 1.4 order, 1.6 order, 1.8 order, and 2.0 order) are plotted in Fig. 3. For the raw reflectance data, 45 bands passed the significance test at 0.01, but as the order of the derivative increases, correlation coefficients increase beyond the 0.01 level in some wavelength ranges. However, band values do not pass the significance test at 0.01. In addition, as the order declines from 1.0 to 2.0, band values increasingly pass the significance test at the 0.01. As correlation coefficients increase, when the order reaches 1.6, correlation coefficients reach 0.68 at 1,368 nm. On the whole, the curves fluctuate greatly, and thus more information cannot be derived from Fig. 3.

**Figure 4:**
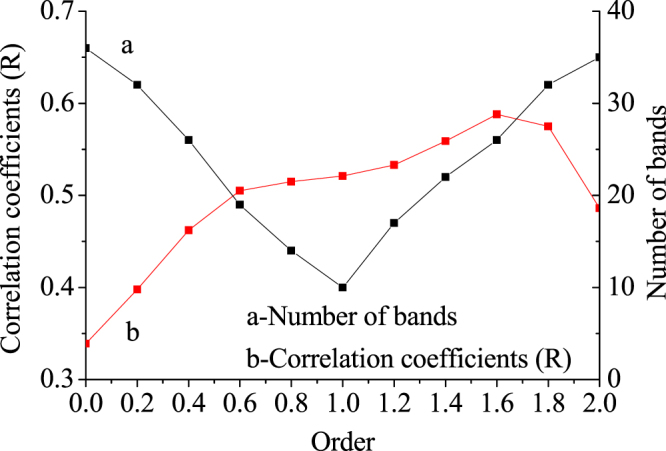
The number of bands passing the significance test and trend lines (Map by Origin 9.1 (http://www.originlab.com/software)).

From Fig. 3 it is not clear how many bands of raw reflectance data treated by fractional derivatives passed the significance test at 0.01, and thus raw reflectance data and raw reflectance data treated by fractional derivatives are measured and corresponding trend lines and relationships between raw reflectance data and raw reflectance data treated by fractional derivatives and the water quality index (WQI) are shown in Fig. 4. For these 11 mathematical forms of reflectance, different numbers of bands passed the significance test. With an increase in derivative order, values first decreased and then increased, and all reached a minimum value at the 1.0 fractional orders and a maximum value at the 1.6 fractional orders. However, band numbers do not pass the significance test at 0.01. In addition, as the order declines from 1.0 to 2.0, band numbers increasingly pass significance testing at 0.01. As correlation coefficients increase, once the order reaches 1.6, the correlation coefficient is 0.68.

### Relationships between the water quality index (WQI) and the spectral indices

**Figure 5:**
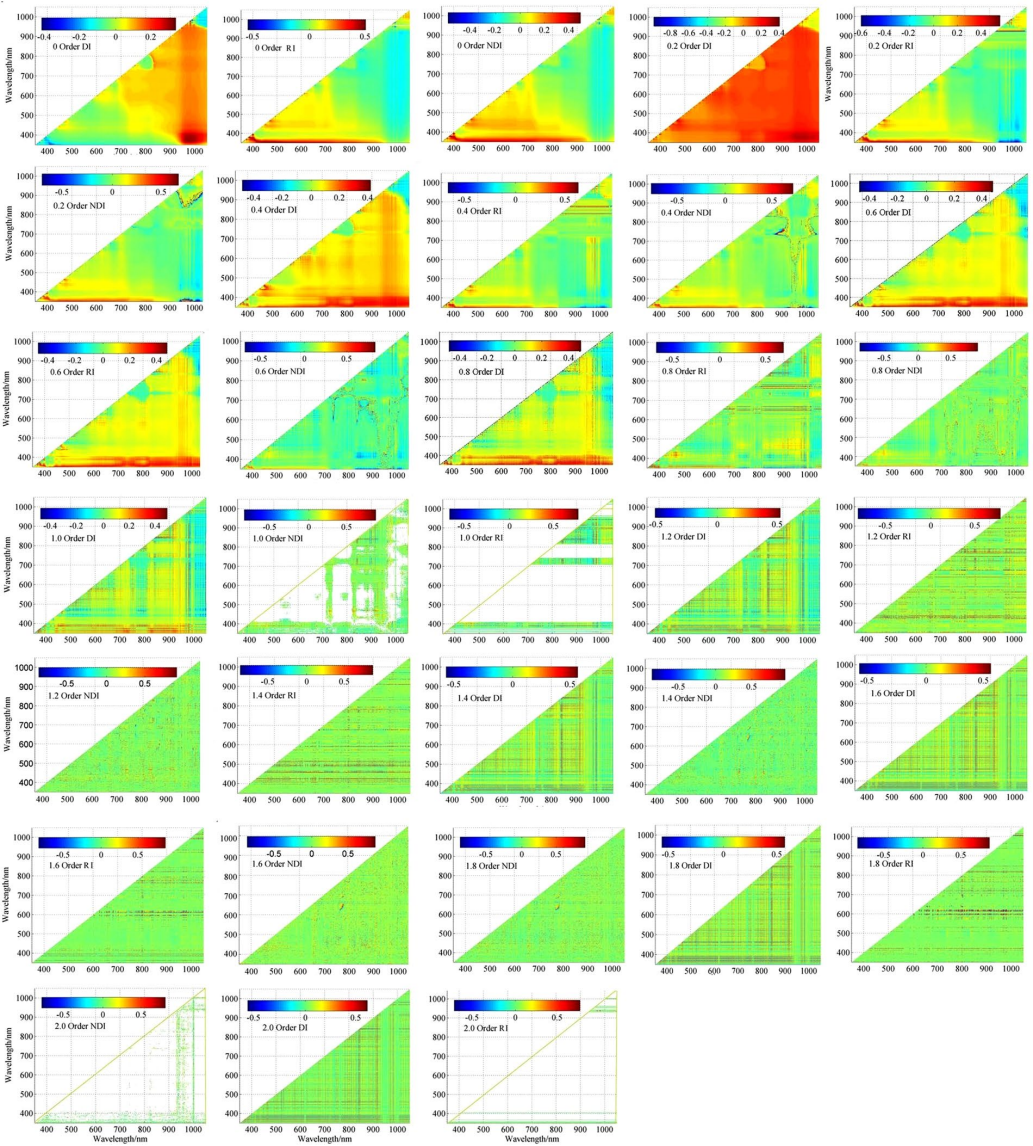
Contour maps of correlation coefficients (r) between WQI values and normalized difference, ratio, and difference spectral indices based on raw reflectance data treated by fractional derivatives using two reflectance spectra at *i* and *j* nm (n = 48). (Map by MATLAB 2014a (https://www.mathworks.com/software)).

Contour maps of *r* values between the water quality index (WQI) and two-band spectral indices (DI, NDI and RI) are shown in Fig. 5. A strong correlation between the DI, NDI and RI and the water quality index (WQI) is largely found in the visible and near-infrared ranges (Fig. 5). While the performance of the three spectral indices as predictors of the water quality index (WQI) appears to vary by wavelength, constant forms are revealed. Wavelength combinations in the 350–1100 nm region for R^2^ spectra (Fig. 5) show a significant correlation between the RI and the water quality index (WQI).

Wave bands of combinations (DI, RI and NDI) for the reflectivity of the raw spectrum curve and raw reflectance data treated by fractional derivatives and corresponding strong correlations with the water quality index (WQI) were mainly found to be concentrated in two zones (Fig. 5). The ratio index (RI) sensitivity region and normalization index sensitivity region were found to be nearly consistent. However, index sensitivity zones were found to differ. For the RI, good wavelength combinations were observed with R_2_ values of 0.40 and 0.92, respectively (Table 3). The correlation *r* is minimal in raw reflectivity wave bands of the combinations (R_883_/R_934_), and the maximum correlation coefficient value is found in raw reflectance data treated by 1.6 order derivatives located at R_600_ − R_900_. For the different index (DI), good wavelength combinations were observed with R_2_ values of 0.497 and 0.585, respectively (Table 3). The lowest correlation *r* is found in raw reflectivity wave bands of the combinations (R_583_ − R_844_), and the maximum correlation coefficient is found in raw reflectance data treated by 1.6 order derivatives for R_500_ to R_900_. For the normalized index (NDI), good wavelength combinations were found with R_2_ values of 0.764 and 0.914, respectively (Table 3). The weakest correlation *r* is found in raw reflectance data treated by 0.2 order derivatives of combinations ((R_520_ − R_760_)/(R_520_ + R_760_)), and the largest correlation coefficient is found in raw reflectance data treated by 1.6 order derivatives in the R_452_ and R_703_ zones. Raw observations show several weak absorption regions at close to 452 and 703 nm, and R_452_ and R_703_ zones of NDI wave bands of the combinations correlation coefficient are the highest. Therefore, the spectrum absorption valley is central to the study of water quality sensitivity levels. In addition, a reflectivity value of 964 nm is found in the most important area of the sensitive band. This analysis reveals the presence of a strong correlation between DI, RI, NDI and the different water quality indices. Strong correlations with water quality are mainly found as *r* values (Table 3).

**Table 3:** Correlation coefficients between WQI and each order derivative of raw spectral reflectance of RI, DI, NDI.

Derivative order	RI		DI		NDI	
	Band	R	Band	R	Band	R
0	R_988_/R_969_	0.4023	R_988_ − R_969_	0.4978	(R_963_ − R_989_)/(R_963_ + R_989_)	0.5917
0.2	R_359_/R_675_	−0.9861	R_359_ − R_1016_	−0.5500	(R_890_ − R_1017_)/(R_890_ + R_1017_)	0.7210
0.4	R_576_/R_954_	−0.6826	R_838_ − R_840_	0.4914	(R_576_ − R_954_)/(R_576_ + R_954_)	0.7983
0.6	R_717_/R_1034_	0.8225	R_843_ − R_844_	0.4826	(R_556_ − R_1131_)/(R_556_ + R_1131_)	0.8314
0.8	R_840_/R_915_	0.7322	R_838_ − R_840_	0.4948	(R_424_ − R_828_)/(R_424_ + R_828_)	0.9057
1.0	R_902_/R_915_	0.7974	R_855_/ − R_844_	0.4952	(R_354_ − R_956_)/(R_354_ + R_956_)	0.8793
1.2	R_652_/R_926_	0.8354	R_840_ − R_846_	0.5242	(R_652_ − R_926_)/(R_652_ + R_926_)	0.9104
1.4	R_359_/R_854_	0.9089	R_622_ − R_844_	0.5807	(R_359_ − R_854_)/(R_359_ + R_854_)	0.9200
1.6	R_883_/R_934_	0.9274	R_583_ − R_844_	0.5811	(R_520_ − R_760_)/(R_520_ + R_760_)	0.9299
1.8	R_463_/R_964_	0.8884	R_465_ − R_844_	0.5744	(R_452_ − R_703_)/(R_452_ + R_703_)	0.9144
2.0	R_463_/R_933_	0.8482	R_969_ − R_988_	0.5118	(R_956_ − R_973_)/(R_956_ + R_973_)	0.8113

### Particle swarm optimization (PSO)-support vector regression model

#### Establishing a WQI estimation model based on a support vector regression model

MATLAB 2014a is applied to design a particle swarm optimization (PSO) support vector regression model. Hyperspectral parameters of sensitive wave bands and the spectral index and water quality index (WQI) of the Ebinur Lake wetlands are used to develop a particle swarm optimization - support vector regression model (POS-SVR). Data were randomly chosen and segregated into training and testing components at a 7:3 ration. After training the model (POS-SVR), it was tested using 30% of the data that differed from the training set. This was conducted to assess the generalization accuracy of the trained model and to ascertain its capacity to use the SVR learned pattern to predict target values for previously unseen datasets. This method is referred to as model validation and the performance assessment method used is only as good as the criteria set for this reason. Each input factor applies a different measurement unit. To eliminate dimension effects of these variables and to realize equivalent expression effects for each input factor, the non-dimensional method is applied for the data analysis to standardize various input factors and to compress the scope of change for each input factor to −1 to 1. The premnmx function is applied in MATLAB 2014a to normalize the input factors. When the nerve cell is satisfactorily accurate, the postmnmx function can be applied to recover the original magnitude of the normalized data. The different input parameters of the POS-SVR model for parameter comparison is as described in Table 4.

**Table 4:** Input parameters of the POS-SVR model for parameter comparison.

Input Parameter	Order	Output Parameter	POS-SVR						
			c	g	mse	R^2^	RMSE	SD	RPD
Single bands	0	WQI	1.6957	0.1000	1.7402	0.80	287.94	484.73	1.68
	0.2	WQI	48.7120	0.0091	1.3434	0.79	306.46	542.08	1.76
	0.4	WQI	32.1190	0.0075	1.3245	0.75	312.75	380.75	1.22
	0.6	WQI	33.1999	0.0097	1.4578	0.88	144.58	328.85	2.27
	0.8	WQI	1. 9675	0.1000	1.7711	0.85	269.48	414.74	1.54
	1.0	WQI	55.712	0.0091	1.8434	0.86	234.65	553.96	2.36
	1.2	WQI	42.197	0.0083	1.2781	0.83	252.26	483.27	1.92
	1.4	WQI	33.1999	0.0097	1.4578	0.87	219.45	545.52	2.49
	1.6	WQI	1. 9675	0.2000	1.7751	0.91	183.91	467.97	2.57
	1.8	WQI	55.712	0.0121	1.8734	0.84	253.19	485.61	1.96
	2.0	WQI	42.197	0.0083	1.2981	0.79	285.43	506.15	1.77
DI, RI, NDI	0	WQI	1.6957	0.1000	1.7902	0.88	201.14	446.82	2.22
	0.2	WQI	48.712	0.0091	1.3434	0.88	214.41	506.22	2.36
	0.4	WQI	32.197	0.0083	1.2781	0.77	296.95	306.11	1.03
	0.6	WQI	88.1235	0.1008	2.1789	0.87	218.99	366.05	1.67
	0.8	WQI	1.6957	0.1090	1.7402	0.72	344.55	386.88	1.12
	1.0	WQI	48.7120	0.0091	1.3434	0.86	233.48	518.12	2.22
	1.2	WQI	32.1190	0.0075	1.3245	0.89	198.62	505.21	2.53
	1.4	WQI	33.1999	0.0097	1.4578	0.89	212.73	441.58	1.08
	1.6	WQI	1. 9675	0.1000	1.7711	0.92	165.91	429.78	2.59
	1.8	WQI	33.1999	0.0097	1.4578	0.86	213.35	484.15	2.26
	2.0	WQI	1. 9675	0.1340	1.2211	0.85	251.15	513.08	2.04

#### Verifying the estimation model of the water quality index

**Figure 6:**
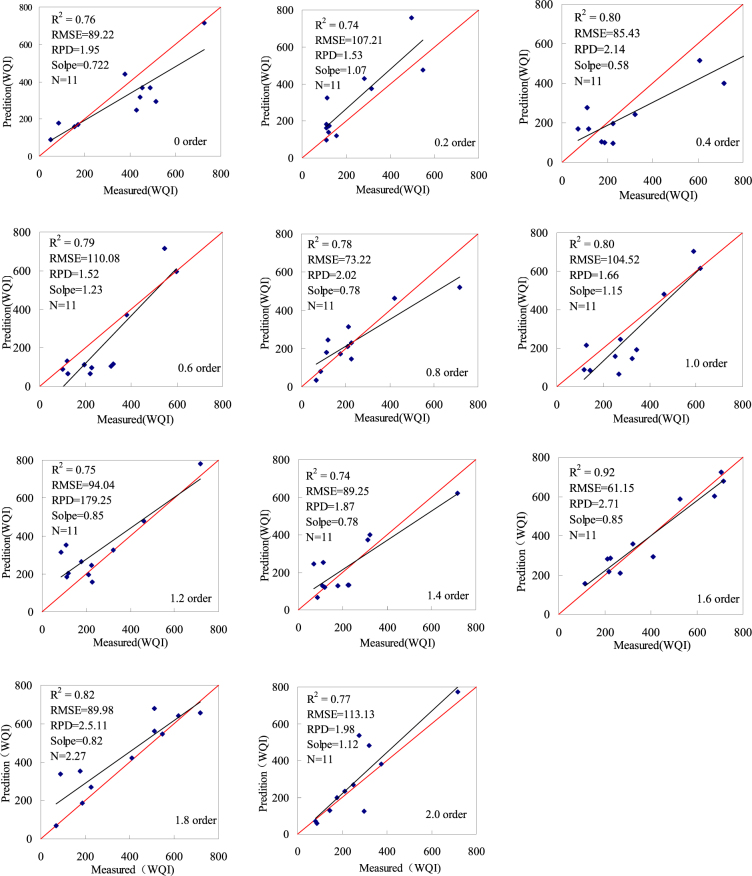
Correlations between the measured verification values and the predicted values based on a sensitivity bandpass significance test conducted at the 0.01 level (Map by EXCEL (https://www.microsoft.com/software)).

**Figure 7:**
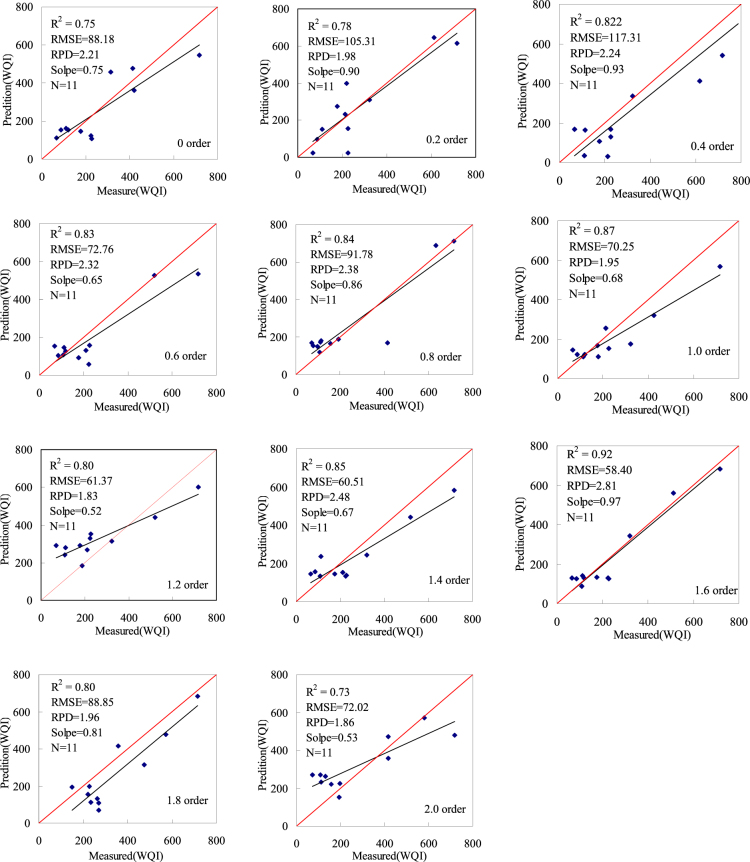
Correlations between the measured verification values and predicted values based on the spectral index (RI, DI, and NDI) (Map by EXCEL (https://www.microsoft.com/software)).

After modeling different water quality indices (WQIs), the accuracy of obtained models was examined for an independent dataset consisting of 11 samples. The corresponding validation results are shown in Figs 6, 7 and statistical results are summarized in Table 5. Scatter diagrams are presented for prediction and real values of the inversion model in Figs 6, 7. The coefficient of determination R^2^ between predicted and measured values for monitoring model accuracy is higher, the measured and predicted values are basically linear, and the RMSE is low while the slope of the fitting curve is closer to 1. Therefore, the related POS-SVR model exhibits a strong non-linear fitting capacity, denoting excellent effects of the hyperspectral spectral index on the monitoring water quality index (WQI). Figures 6, 7 and Table 5 show a scatter diagram for the measured real and predicted values.

**Table 5:** Summary of parameter correlations between the measured verification values and predicted values.

X	Order	Y	GA-SVR					
			R^2^	RMSE	SD	RPD	Slope	N
Single bands	0	WQI_P_	0.76	89.22	174.23	1.95	0.72	11
	0.2	WQI_P_	0.74	107.21	163.99	1.53	1.07	11
	0.4	WQI_P_	0.80	85.43	182.83	2.14	0.58	11
	0.6	WQI_P_	0.79	110.08	167.21	1.52	1.23	11
	0.8	WQI_P_	0.78	73.22	148.26	2.02	0.78	11
	1.0	WQI_P_	0.80	104.52	173.07	1.66	1.15	11
	1.2	WQI_P_	0.75	94.04	179.25	1.91	0.85	11
	1.4	WQI_P_	0.74	89.25	167.33	1.87	0.78	11
	1.6	WQI_P_	0.92	61.15	166.22	2.71	0.85	11
	1.8	WQI_P_	0.82	89.98	205.11	2.27	0.82	11
	2.0	WQI_P_	0.77	113.13	224.73	1.98	1.12	11
RI,DI,NDI	0	WQI_P_	0.75	88.18	194.81	2.21	0.75	11
	0.2	WQI_P_	0.78	105.31	209.37	1.98	0.90	11
	0.4	WQI_P_	0.82	117.31	263.79	2.24	0.93	11
	0.6	WQI_P_	0.83	72.76	169.05	2.32	0.65	11
	0.8	WQI_P_	0.84	91.78	218.52	2.38	0.86	11
	1.0	WQI_P_	0.87	70.25	137.32	1.95	0.68	11
	1.2	WQI_P_	0.80	61.37	112.31	1.83	0.52	11
	1.4	WQI_P_	0.85	60.51	149.89	2.48	0.67	11
	1.6	WQI_P_	0.92	58.40	164.16	2.81	0.97	11
	1.8	WQI_P_	0.80	88.85	174.52	1.96	0.81	11
	2.0	WQI_P_	0.73	72.02	133.63	1.86	0.53	11

Figures 6, 7 and Table 5 show that the predicted water quality index (WQI) value is very consistent with the measured water quality index value. The 15 water quality index estimation models were validated by the 22 water samples. In total, 22 models present acceptable results at RPD > 1.4 and with a slope of close to 1. The sensitive wave band estimation model is more accurate for the 1.6 order derivates. R^2^ is valued at 0.92; RMSE is valued at 58.40, RPD is valued at 2.71, and the slope is valued at 0.85. The spectral index estimation model is more accurate for the 1.6 derivates. R^2^ is valued at 0.92; RMSE is valued at 61.15, RPD is valued at 2.81, and the slope is valued at 0.97.

#### Compare the accuracy of the machine learning algorithm and geographically weighted regression (GWR)

**Figure 8:**
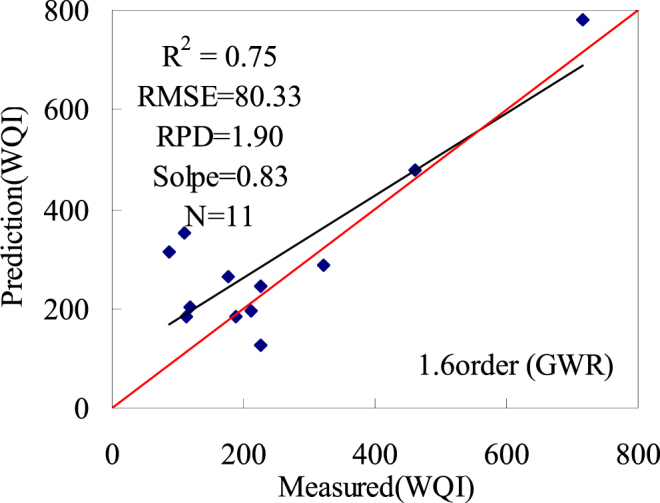
Scatter plot of measured and predicted WQI in GWR models (Map by EXCEL (https://www.microsoft.com/software)).

R_883_/R_934_, R_583 _− R_844_, and (R_520 _− R_760_)/(R_520_ + R_760_) is the independent variable, the GWR model was used for regression analysis of WQI, AIC value is 402.69, R^2^ is 0.86, residual sum of squares value is 879.91. Test the model with a validation sample, R^2^ is 0.75, RMSE is 80.33, and RPD is 1.90. Scatter diagrams are presented for prediction and real values of the inversion model Fig. 8.

Compare the accuracy of the machine learning algorithm and geographically weighted regression (GWR), the spectral index estimation model is more accurate for the 1.6 derivates based on machine learning algorithm. R^2^ is valued at 0.92; RMSE is valued at 61.15, RPD is valued at 2.81, and the slope is valued at 0.97. Therefore, the water quality index (WQI) monitoring model based on machine learning algorithm is highly stable and presents a high level of predictive capacity. The Particle swarm optimization - support vector regression model can thus be used to generate water quality index estimations for the semi-arid central Asian zone of Xinjiang, China.

## Discussion

Assessment of water quality and of the spatial variability of the water quality index (WQI).

In this study, the water quality of Ebinur Lake watershed surface water was evaluated. Rivers of the Ebinur Lake Watershed recharge Ebinur Lake. To evaluate the water quality levels of Ebinur Lake Watershed surface water, 48 sampling sites and 20 water quality parameters were selected for monitoring and analysis. Water quality parameters pH, HCO_3_^2−^, TP, TN, BOD_5_, NH_3_^+^-N, Iron, Copper, Zinc, Volatile phenol, DO, TDS, Cl^−^, SO_4_^2−^, Na, Ca, Mg, COD, PO_4_^3−^ and Cr were used to calculate WQI values to evaluate river water quality levels. WQI values were found to range between 56.61 and 2886.52. The WQI classification shows that the Ebinur Lake Watershed presents varying levels of water quality. The downstream areas of the river present poor water quality levels, where the main pollutant sources include wastewater discharged from Wenquan County and the city of Bole, leather and marble factories downstream from the Boertala River Valley and agricultural activities in the oasis of the Ebinur Lake Watershed; the main pollutant sources include wastewater discharged from Jinghe County, the leather industry, saltwork and saline land downstream from the Jinghe River and agricultural and grazing activities in the oasis of the Ebinur Lake Watershed. The Kuitun-Akeqisu River is located in the southwestern area of the watershed. A large amount of salt is found on either side of the river, and water quality in the area is highly saline. Effects of water quality parameters on the WQI map were investigated. Consequently, environmental pollutants negatively affect all water surfaces of the Ebinur Lake Watershed. Therefore, necessary protection measures should be taken on the planned usage of river water.

### Estimate water quality index (WQI) value based on hyperspectral remote sensing data

In this study, an estimated water quality index (WQI) value is established based on sensitive wave bands and a spectral index of hyperspectral data. Water quality levels are directly estimated and assessed via remote sensing techniques. Most previous studies^18,34,35^ have focused on single indices of water quality such as chlorophyll-a, TDS, and NTU. While single indices of water quality are monitored using remote sensing technologies, and while single water quality parameters of monitoring models are highly accurate, such results are uncertain. As water quality conditions are reflected by all water quality parameters, overall water quality conditions are monitored by remote sensing; spectral reflectance values reflect overall parameters. Therefore, single indices of water quality monitored using remote sensing technologies are uncertain. The water quality index (WQI) reflects overall water quality conditions. The evaluation and estimation of surface water quality based on the hyperspectral remote sensing is feasible. In this study the accuracy of the estimation model is improved through the use of new hyperspectral indices (DI, RI, and NDI) and via particle swarm optimization - support vector regression. Remote sensing techniques make it possible to develop a spatial and temporal understanding of surface water quality indices and to more effectively and efficiently monitor water surfaces. Such tools can also be used to estimate water quality distributions. Future studies must measure the applicability of satellite remote sensing data and of unmanned aerial vehicle (UAV) technologies for estimating WQI values. As the number of *in situ* samples continues to increase, a unique regression model that effectively measure the water quality parameters of different watersheds should be developed for arid regions.

## Conclusions

The Ebinur Lake Watershed of the Xinjiang Autonomous Region, China, was used as a study area. We used optimal bands based on difference index, ratio index, and normalized difference index algorithms to assess the WQI using spectral eleven orders (interval 0.2) of fractional derivatives for remote sensing data, and we measured the performance of the proposed models using GA-SVR and the band difference algorithm. The results are as follows:

1. Water quality levels for drinking purposes were evaluated via the water quality index (WQI) method. The computed WQI values were found to range between 56.6133 and 2,886.5198. The prepared WQI map shows that the arid area generally presents low levels of water quality.
2. As the order increased, the number of bands with correlation coefficients passing a significance test at 0.01 first increased and then decreased with a peak appearing with the 1.6 order and with an R^2^ of 0.525. The WQI and derivative spectral data of DI, RI and NDI correlation coefficients among the optimal band combinations also show a peak with the 1.6 order and R^2^ values of 0.818, 0.8624 and 0.8297.
3. In total, 22 WQI estimation models were generated from a principal component single band and from RI, DI, and DNI values based on the 1.6 order derivative, the lowest RMSE, the highest R^2^ (0.92) and the RPD (2.59).
4. Comparisons of the predictive effects of the 22 water quality index estimation models calibrated by POS-SVR show that the model based on RI, DI, and NDI values of the 1.6 order is much better than the others while better predicting the water quality index of the study area (R^2^ (0.92), RMSE = 58.4, RPD (2.81) and a slope of curve fitting of 0.97).

This study not only estimates a water quality index using different techniques for the semi-arid area of central Asia but also develops a new algorithm that can be applied to this area and to other areas.

## Materials and Methods

### Study area

**Figure 9:**
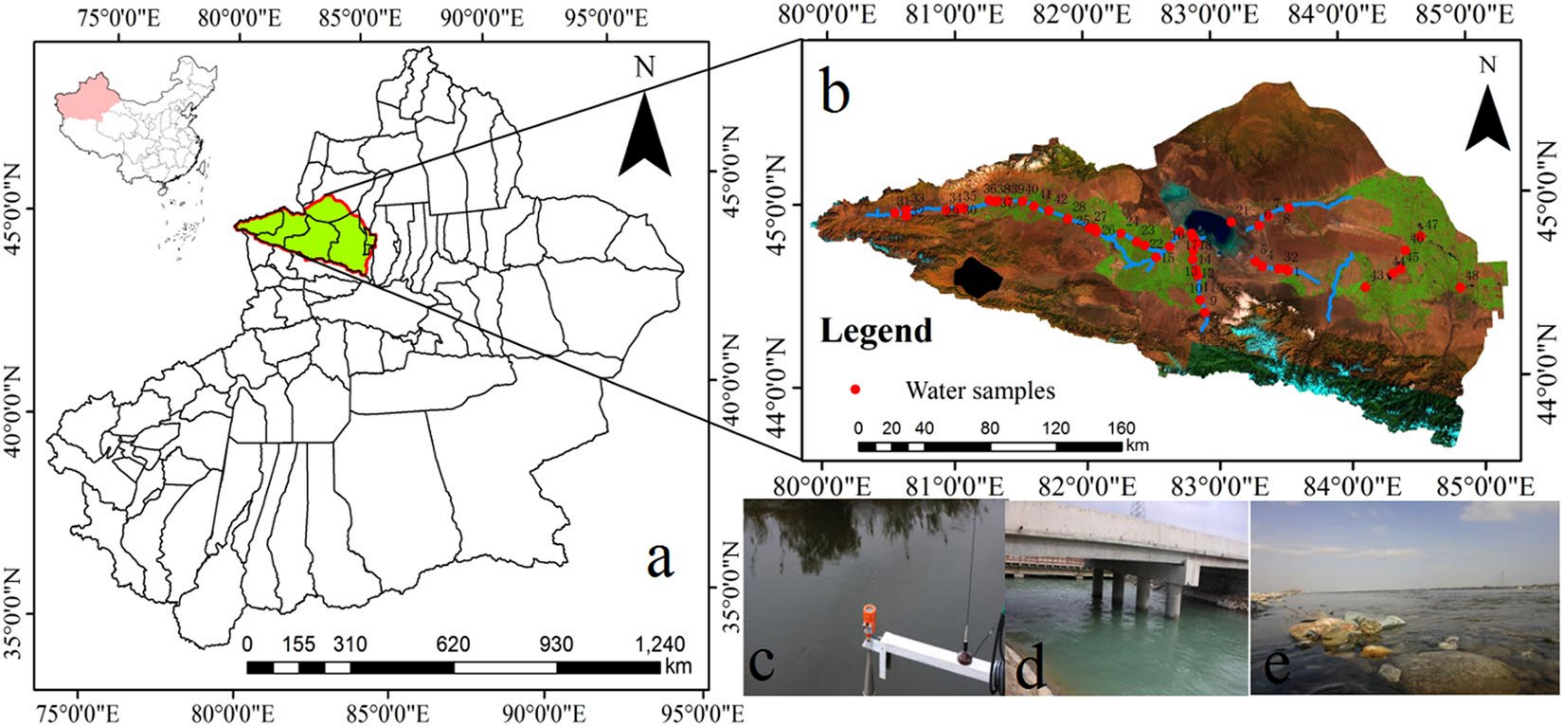
(**a**) Map of the study area with an inset map showing the location of the Xinjiang Autonomous Region within China; (**b**) satellite map of the study area; (**c**) Kuitui River, (**e**) Boertala River, (**e**) Jing River; photographs of the three selected sampling locations (photographed by Xiaoping Wang, Map by ArcGIS10.2.2 (http://www.esri.com/software/arcgis)).

The Ebinur Lake Watershed (44°05′−45°08′N, 82°35′−83°16′E) (Fig. 9) is located on the northern slope of the Tien Shan Mountains southwest of the Junggar Basin. The watershed covers an area of 50,621 km^2^. It is surrounded by a mountainous region (24,317 km^2^; Alatau Mountains, Maliyi Mountains and Biezhentao Mountains) and by plains (Jinghe Oasis) (26,304 km^2^) to the north, west and south^36^. Artificial reservoirs (RES) are found southwest of the watershed. The area is characterized by a typical temperate arid continental climate with the mountain-oasis-desert system presenting typical temperate arid ecological characteristics. The study region is located inland (2,000 km from the Pacific and Indian Ocean and 3,000 km from the Arctic Ocean); moisture in the study area is derived from the Atlantic Ocean (7,000 km), but water vapor transport from maritime areas is limited^36^.

The lake is a terminal lake fed by the Kuitun Mountains, Akeqisu River, Jing River, Tuotuo River, Sikeshu River, Boertala River, Akaer River and Daheyanzi River. Surface water levels of Ebinur Lake and the Tuotuo River are currently low and thus water ecological safety levels are threatened. Severe water shortage problems and the presence of large volumes of sewage have rendered river and lake water pollution levels high in the Ebinur Lake Watershed, a typical arid area of central Asia^37^.

## Materials

### Sample collection

Water samples were collected on October 5, 2016 from 48 locations within the Ebinur Lake Watershed (Fig. 9). Collected water quality samples were stored at low temperatures (under 2 °C) during transport before water quality measurements were carried out in a laboratory. Samples were transported in polyethylene plastic bottles previously rinsed with 10% HCI and cleaned with deionized water to minimize changes in water chemical characteristics. We used a handheld global positioning system (GPS) indicator to determine the central coordinates of each sample and used a digital camera to photograph the sampling area (see Fig. 9). Temperature and pH levels were recorded at the time of sampling along the shore. All other measurements were taken within a day following sample collection in the lab. Biochemical oxygen demand (BOD_5_), total nitrogen (TN), total phosphorus (TP), iron, copper, chemical oxygen demand (COD), zinc, volatile phenol (V.P.) ammonia nitrogen (NH_3_^+^-N), Henderson-Hassebalch (HCO_3_^−^), dissolved oxygen (DO), total dissolved solids (TDS), chloride (Cl^−^), sulphate ion (SO_4_^2−^_)_, natriumion (Na), calcium (Ca), magnesium (Mg), phosphate (PO_4_^3−^) and (Chromium VI) Cr concentrations collected over five days were determined according to corresponding methods as is shown in Table 6.

**Table 6:** Water indices and experimental methods.

	Water quality indices	Experimental methods
1	DO	According to the iodine quantity method (GB/7489–7489), we used a visible light spectrophotometer 722 N test instrument to measure DO levels.
2	COD	According to the dichromate method (GB 11914–1989), we used a standard COD digestion apparatus (K-100) to determine COD levels.
3	BOD_5_	We used the dilution and inoculation method (HJ 505–2009) and a constant temperature incubator (HWS-150 type) to measure BOD_5_ content levels.
4	TP	Using the ammonium molybdate spectrophotometric method (HJ 636–2012), we employed a visible light spectrophotometer 722 N to determine TP content levels.
5	TN	Via ultraviolet spectrophotometry (HJ 535–2009), we used an ultraviolet visible light spectrophotometer and UV-6100 to determine TN content levels.
6	NH_3_^+^-N	Using Nessler’s reagent spectrophotometer and a visible light spectrophotometer 722 N for the determination of NH_3_^+^-N levels.
7	pH	pH-40A portable pH acidity meter.
8	Iron	According to atomic absorption Spectrophotometer methods
9	Copper	According to atomic absorption Spectrophotometer methods
10	Zinc	According to atomic absorption Spectrophotometer methods
11	Volatile phenol	The direct photometric amino antipyrine method was used to measure volatile phenol
12	TDS	The WTW inoLab^@^ Multi 3420 Set B multi-parameter measurement instrument (Wissenschaftlich-Technische Werkstätten GmbH, Germany) was used.
13	Ca	Atomic absorption spectrometry methods were used.
14	Mg	According to atomic absorption Spectrophotometer methods
15	Na	Sodium ion electrode methods were used.
16	Cl^−^	Silver nitrate titration methods (GB T5750.5–2006) were used.
17	HCO_3_^−^	Drop-counting microtitrimetry methods (SL83–94) were used.
18	SO_4_^2−^	Methylene blue methods (GB T5750.5–2006) were used.
19	PO_4_^3−^	Phosphorus molybdenum blue colorimetric methods (GB T5750.5–2006) were used.
20	Cr	Diphenylcarbazide photometry methods (GB T5750.5–2006) were used.

### Hyperspectral data collection

The FieldSpec^③^3 ASD Spectroradiometer device is an optical sensor that uses detectors other than photographic film to measure the distribution of radiation in a particular wavelength region to measure the radiant energy level (radiance and irradiance). It was used to visualize spectral reflectance patterns of lake water corresponding to water content levels. Observation methods applied to water surfaces can be found in Supplementary Fig. S1.

To observe the water surfaces (Fig. S1), the spectral range of the spectrometer was set to 350–1050 nm with a 1 nm sampling interval. To avoid environment changes in illumination conditions, measurements between water the target, sky, and whiteboard were collected at each station. Sky conditions were also recorded at each station during spectral measurement.

All field spectrometer measurements were processed to remove sky and sun glare using a constant water body reflection coefficient^38^. Therefore, hyperspectral reflectance values, R_rs_, were calculated using the following equation:

1 $${R}_{rs}=\frac{{L}_{u}-\rho {L}_{s}}{{E}_{d}}$$

where *L*_*u*_ is the total upwelling radiance, *L*_*s*_ is the sky radiance*, ρ* is the water surface reflection efficiency level of 0.028, an_*d*_ *E*_*d*_ is the measured down welling solar irradiance.

## Methods

### Fractional Derivative Method

Fractional derivative methods have been widely used in certain fields because models described by the fractional derivative are more accurate and efficient than methods based on integer derivatives^39,40^. The most frequently used definitions are the following: Grunwald - Letnikov (G-L), Riemann - Liouville (R-L), and Caputo^41^. As it is less complex than the others, the G-L definition was employed in this study. Grunwald - Liouville is defined as follows^42^:

2 $${d}^{a}f(x)=\mathop{\mathrm{lim}}\limits_{h\to 0}\frac{{\rm{1}}}{{h}^{a}}\sum _{j=0}^{[(t-a)]}{(-1)}^{j}(\frac{a}{j})\,f(t-jh)$$

where *a* is the step length, where *h* is the order number, and where *t* and *a* are the respective upper and lower limits of the derivative. The Gamma formula is written as follows:

3 $$\Gamma (a)={\int }_{0}^{\infty }exp(-u){u}^{a-1}du=(a-1)!.$$

Based on our use of ASD spectrometer data, when the sampling interval is 1 nm, *h* = 1. *f*_*(X)*_ is the fractional order derivative, which is defined as follows:

4 $$\frac{{d}^{a}f(x)}{d{x}^{a}}\approx f(x)+(-a)f(x-1)+\frac{(-a)(-a+1)}{2}f(x-2)+\mathrm{......}+\frac{\Gamma (-a+1)}{n!\Gamma (-a+n+1)}f(x-n).$$

Therefore, (5) can be regarded as the numerical algorithm used to calculate the fractional derivative of hyperspectral data, and a zero order denotes that hyperspectral data are not treated by the derivative algorithm.

### Determination of the best indices

In obtaining the most sensitive bands from water environment data, previous studies show that the combination of various bands can improve the sensitivity of hyperspectral reflectance data to water quality values^43^. Therefore, this method explores the relationships between water quality and the spectrum reflectance and then applies a 2D correlation diagram to study relationships between the difference index (DI), ratio index (RI), normalization index (NDI), and water quality index^44^. Optimal combination bands for the water quality index value are selected from 350 nm-1,050 nm and are entered into MATLAB 2014a (MathWorks, 2014).

5 $${\rm{DI}}({{\rm{R}}}_{{\rm{i}}},\,{{\rm{R}}}_{{\rm{j}}})\,=\,{{\rm{R}}}_{{\rm{i}}}{-R}_{{\rm{j}}}$$

6 $${\rm{NDI}}({{\rm{R}}}_{{\rm{i}}},{{\rm{R}}}_{{\rm{j}}})={(R}_{{\rm{i}}}\,-\,{{\rm{R}}}_{{\rm{j}}}{)/(R}_{{\rm{i}}}+{{\rm{R}}}_{{\rm{j}}})$$

7 $${\rm{RI}}({{\rm{R}}}_{{\rm{i}}},{{\rm{R}}}_{{\rm{j}}})={{\rm{R}}}_{{\rm{i}}}/{{\rm{R}}}_{{\rm{j}}}$$

R_i_ and R_j_ are random bands selected at 350 nm –1,050 nm while R_i_ and R_j_ denote the original reflectivity values of any two bands selected at 350 nm–1,050 nm.

### Calculation of the Water Quality Index (WQI)

The Water Quality Index (WQI) is an extracted and estimated index that reflects the composite effects of all water quality parameters^45^. First, each water quality parameter was assigned a weight (W_i_) from a scale of 1 (lowest effect on water quality parameters) to 5 (strongest effect on water quality parameters) based on perceived effects on primary health and according to its relative importance to the surface water environment^46,47^. PO_4_^3−^, SO_2_ and Cr values were assigned the highest weight (8) due to their primary role in water quality assessments; a minimum weight of 1 was assigned to parameters Ca, Mg and Na due to their limited importance for water quality assessments^48^. The relative weight (W_i_) is computed from the following equation:

8 $$Wi=\frac{Wi}{{\sum }_{n=1}^{n}Wi}$$

where W_i_ is the relative weight, W_i_ is the weight of each parameter, and *n* is the number of parameters. Then, a quality rating (Q_i_) for each parameter is assigned by dividing its concentration in each water sample by its limit given in the WHO^33^ quality standards for surface water quality for the People’s Republic of China. This result is multiplied by 100;

9 $${Q}_{i}=\frac{{C}_{i}}{{S}_{i}}\times 100$$

where Q_i_ is the quality rating, C_i_ is the concentration of each water quality parameter for each water sample, and S_i_ is the surface water standard for each water quality parameter according to WHO guidelines^33^ (2008). To measure the WQI, the SI_i_ value should be calculated first using the following equations;

10 $$S{I}_{i}={W}_{i}\times {q}_{i}$$

11 $$WQI={\sum }_{i=1}^{n}S{I}_{i}$$

where SI_i_ is the water quality index of the *i*th parameter and Q_i_ is the water quality level based on the *i*th water quality parameter^49^.

### Estimate the WQI using a machine learning algorithm

Machine learning algorithms have become very popular in the era of big data. Machine learning is an artificial science. The field’s main objects of study are artifacts and specifically algorithms that improve performance with experience. The Support Vector Regression (SVR) Model is the main algorithm used for machine learning. We used the Support Vector Regression Model to estimate the WQI for the arid area^50–52^.

Given sample data (x_i_, y_i_), i = 1, 2, ^…^, *l* where x_i_ denotes the input vector,${y}_{i}=f({x}_{i})$ is the estimated output measure. Estimated methods can be written as:$f(x)=\omega \varphi (x)+b$ where $\varphi (x)$ is a nonlinear model drawn from the input space to a high dimensional space; *ω* is a weight vector; and b is the offset.

The regression target identifies parameters $\omega$ and b, which minimize the regression error function. The regression error function can be defined as:

12 $${R}_{reg}(f)=C\sum _{i=1}^{l}\Gamma (f({x}_{i})-{y}_{i})+\frac{1}{2}{\Vert \omega \Vert }^{2}$$

where $\Gamma (.)$ is a loss function and where Constant C > 0 is a fixed penalty parameter. The most commonly used loss function is the $\varepsilon$-insensitive loss function:

13 $${L}^{6}(x,y,f)={|y-f(x)|}_{6}=\,\max (0,|y-f(x)|-\varepsilon )$$

This shows that the loss is 0 when the difference between the measured and predicted value is less than a small positive number of $\varepsilon$. To smooth the regression function, a minimum $\omega$ must be found, and based on the fitting error, the regression function can be solved as a constrained optimization problem:

$$\mathop{{\rm{minimize}}}\limits_{\omega ,b,{\xi }_{i},{\xi }_{j}}\,\frac{1}{2}{\Vert \omega \Vert }^{2}+C\,\sum _{i=1}^{l}({\xi }_{i}+{{\xi }_{j}}^{\ast })$$

14 $${\rm{Subject}}\,{\rm{to}}\,\{\begin{array}{c}{y}_{i}-[{\omega }^{T}\phi ({x}_{i})+b]\le \varepsilon +{\xi }_{i}\\ \left[{\omega }^{T}\phi ({x}_{i})+b\right]-{y}_{i}\le \varepsilon +{\xi }_{i}\\ {\xi }_{i},{\xi }_{j}\ge 0,i=1,2,\mathrm{....},\,l\end{array}$$

where ${\xi }_{i}$ i and ${{\xi }_{j}}^{\ast }$ are slack variables of upper and lower constraints on outputs of the system. The dual optimization problem illustrated in Equation (14) leads to a quadratic programming (QP) solution involving the Lagrange optimization method that can be expressed as:

$$\mathop{{\rm{maximine}}}\limits_{{a}_{i},{a}_{i}}-\frac{1}{2}\sum _{i,j=1}^{l}({a}_{i}-{{a}_{i}}^{\ast })({a}_{j}-{a}_{j})(\phi ({x}_{i})\bullet \phi (x))-\varepsilon \sum _{i=1}^{l}({a}_{i}+{{a}_{i}}^{\ast })+\sum _{i=1}^{l}yi({a}_{j}+{{a}_{j}}^{\ast })$$

15 $${\rm{subject}}\,{\rm{to}}\{\begin{array}{c}\sum _{i=1}^{l}({a}_{i}-{{a}_{i}}^{\ast })=0\\ 0\le {a}_{i},{{a}_{j}}^{\ast }\le C\end{array}$$

where a_i_, a^i^* are Lagrange multipliers. After solving the optimization problem, denote the optimal solution as $\overline{a}={(\overline{{a}_{1}},\overline{{a}_{2}},\overline{{a}_{3}},\mathrm{.....}\overline{{a}_{l}},\overline{{a}_{l}})}^{T},\overline{b}$ and obtain the regression result:

16 $$f(x)=\sum _{i=1}^{l}\overline{{a}_{i}}-\overline{{a}_{i}})(\phi (x))+\overline{b}$$

According to the Hilbert-Schmidt theorem, the inner product $\phi ({x}_{i})\bullet \phi (x)$ can be replaced by a kernel function K(x i, x) that satisfies Mercer’s conditions^53^. Then, the outcome can be rewritten as:

17 $$f(x)=\sum _{i=1}^{l}\overline{{a}_{i}}-{\overline{{a}_{i}}}^{\ast })k({x}_{i},x)+\overline{b}$$

the most commonly used kernel function is the radial basis kernel function (RBF) $K(x,{x}_{i})=\exp (-{\Vert x-{x}_{i}\Vert }^{2}/{\sigma }^{2})$. Three parameters including the penalty coefficient C, the parameter of the kernel function $\sigma$ and the width of the insensitive loss function $\varepsilon$ constitute the model parameters and have a considerable impact on the performance of the SVR model. These parameters are often used by trial and error and are difficult to use to obtain the optimal value. The PSO can extract the optimal value fast in parallel with a complicated search space^54^, and we adopt it to select optimal parameters of the SVR model. The PSO uses particle populations corresponding to individuals in an evolutionary algorithm to explore the solution space of a problem^55,56^. A flowchart for the proposed PSO-SVR algorithm can be found in Supplementary Fig. S2.

### Statistical analysis

Test data analyses were constructed using Origin8.0 (Origin Lab Corporation, America), and Matlab 2014a (Math Works Corporation, America) was applied to design the program environment. The significance of the statistical correlations was evaluated from *P* values and was compared to predicted and measured values from three indices, i.e., the estimate corresponds to high values of R^2^, to the root mean standard error (RMSE) and to the average standard error (SD)^57^ as follows:

18 $${R}^{2}=(\frac{\sum _{i=1}^{N}({x}_{i}-\overline{x})({y}_{i}-\overline{y})}{{\sum }_{i=1}^{N}({x}_{i}-\overline{x})+{\sum }_{i=1}^{N}(y-\overline{y})})$$

19 $$RMSE=\sqrt{\frac{{\sum }_{i=1}^{N}({y}_{i}-{x}_{i})}{N}}$$

20 $$SD=\sqrt{\frac{{\sum }_{i=1}^{N}\overline{V}{({y}_{i})}^{2}}{N}}$$

21 $$RPD=\frac{SD}{RMSE}$$

In formulas (4), (5), (6), and (7), *(x_i_) is the predicted value; (y_i_) is the measured value; N is the total number of samples; $\mathop{x}\limits^{-}$ is the average value of the sampled value, and $\mathop{y}\limits^{-}$ is the average sample forecast value. SD is the standard deviation of the dataset, RMSE is the root mean square error, and when the RMSE is smaller the model’s predictive capacity is stable. As the R^2^ of the decision coefficient approaches a value of 1, the accuracy of the model improves. For a high RPD of the relative analysis error (RPD < 1.4), the model is not reliable. As 1.4 < RPD < 2, the model is moderately accurate, and RPD > 2, the model presents a high level of predictive ability.

Besides *R* ^2^, *RMSE*, *SD* and *RPD*, in order to acquire the accuracy of the estimate model of WQI based on machine learning algorithm, geographically weighted regression (GWR) (http://gwr4.software.informer. com/ download/) model is selected in this study. As highlighted in the literature^58,59^, the main contribution of the GWR technique is the ability to explore the spatial variation of explanatory variables in the model, where the coefficients of explanatory variables may vary significantly over geographical space. Compare and analyze the accuracy of the machine learning algorithm and geographically weighted regression (GWR) model. Verify the reliability of the machine learning algorithm model.

### Water quality assessment standards

The calculated WQI values are classified into five categories as follows^32^. When the WQI value > 50, the water quality level is excellent and is suited for drinking, and values of 50 > and > 100 denote that water quality levels are good. Values of 100 > HIX > 200 denote poor water quality levels. When 200 > HIX > 300, water quality levels are very poor. A value of HIX < 300 denotes that water is unsuitable for drinking (see Table 7).

**Table 7:** Water Quality Index scale.

Class	Threshold value	Water quality
I	>50	Excellent water
II	50–100	Good water
III	100–200	Poor water
IV	200–300	Very poor water
V	>300	Unsuitable for drinking

## Electronic supplementary material

Supporting information
