# Supplementary material for: Evaluation of water quality based on a machine learning algorithm and water quality index for the Ebinur Lake Watershed, China
Source: Sci Rep. 2017 Oct 9;7:12858. doi: 10.1038/s41598-017-12853-y (PMC5634425; doi:10.1038/s41598-017-12853-y)
Supplement: Supplementary file 1 — Supporting information [file 41598_2017_12853_MOESM1_ESM.doc]

**Supplementary material**

**Evaluation of water quality based on a machine learning algorithm and water quality index for the Ebinur Lake Watershed, China**

**Xiaoping WANG**^1, 2^**, Fei ZHANG^1^**^, 2, 3^ **Jianli DING[[1]](#footnote-2)**^, 2, 3^

^1^. College of Resources and Environment Science, Xinjiang University, Urumqi, 830046 Xinjiang; China

^2^. Key Laboratory of Oasis Ecology, Xinjiang University, Urumqi, 830046 Xinjiang, China

^3^. Key Laboratory of Xinjiang wisdom city and environment modeling Urumqi, 830046 Xinjiang, China

Corresponding author: Fei ZHANG Tel:13579925126 E-mail:zhangfei3s@163.com

**Supporting information legends**

**Fig. S1|** Observation methods on the surface of the water (Map by Visio (https://www.microsoft.com/software))

**Fig. S2|** Flow chart of the proposed PSO-SVR algorithm (Map by Visio (https://www.microsoft.com/software))

**Fig. S1** Observation methods on the surface of the water (Map by Visio (https://www.microsoft.com/software))

**Fig. S2** Flow chart of the proposed PSO-SVR algorithm (Map by Visio (https://www.microsoft.com/software))

1. [↑](#footnote-ref-2)
